# New Bacteriophages against Emerging Lineages ST23 and ST258 of *Klebsiella pneumoniae* and Efficacy Assessment in *Galleria mellonella* Larvae

**DOI:** 10.3390/v11050411

**Published:** 2019-05-03

**Authors:** Damien Thiry, Virginie Passet, Katarzyna Danis-Wlodarczyk, Cédric Lood, Jeroen Wagemans, Luisa De Sordi, Vera van Noort, Nicolas Dufour, Laurent Debarbieux, Jacques G. Mainil, Sylvain Brisse, Rob Lavigne

**Affiliations:** 1 Bacteriology, Department of Infectious and Parasitic Diseases, FARAH and Faculty of Veterinary Medicine, ULiège, 4000 Liège, Belgium; jg.mainil@uliege.be; 2 Biodiversity and Epidemiology of Bacterial Pathogens, Institut Pasteur, 75015 Paris, France; virginie.passet@pasteur.fr (V.P.); sylvain.brisse@pasteur.fr (S.B.); 3 Laboratory of Gene Technology, Department of Biosystems, KU Leuven, 3001 Heverlee, Belgium; danis@wp.pl (K.D.-W.); cedric.lood@kuleuven.be (C.L.); jeroen.wagemans@kuleuven.be (J.W.); rob.lavigne@kuleuven.be (R.L.); 4 Centre of Microbial and Plant Genetics, Department of Microbial and Molecular Systems, KU Leuven, 3001 Heverlee, Belgium; vera.vannoort@kuleuven.be; 5 Department of Microbiology, Institut Pasteur, 75015 Paris, France; luisa.de_sordi@sorbonne-universite.fr (L.D.S.); nicolas.dufour@ght-novo.fr (N.D.); laurent.debarbieux@pasteur.fr (L.D.); 6 Laboratoire des Biomolécules, Hôpital Saint-Antoine, Sorbonne Université, 75012 Paris, France; 7 Institute of Biology Leiden, Leiden University, 2311 Leiden, The Netherlands; 8 Service de Réanimation Médico-Chirurgicale, Centre Hospitalier René Dubos, 95300 Pontoise, France

**Keywords:** antimicrobial resistance, capsule, *Galleria mellonella*, *Klebsiella pneumoniae*, phage therapy

## Abstract

*Klebsiella pneumoniae* is a bacterial pathogen of high public health importance. Its polysaccharide capsule is highly variable but only a few capsular types are associated with emerging pathogenic sublineages. The aim of this work is to isolate and characterize new lytic bacteriophages and assess their potential to control infections by the ST23 and ST258 *K. pneumoniae* sublineages using a *Galleria mellonella* larvae model. Three selected bacteriophages, targeting lineages ST258 (bacteriophages vB_KpnP_KL106-ULIP47 and vB_KpnP_KL106-ULIP54) and ST23 (bacteriophage vB_KpnP_K1-ULIP33), display specificity for capsular types KL106 and K1, respectively. These podoviruses belong to the *Autographivirinae* subfamily and their genomes are devoid of lysogeny or toxin-associated genes. In a *G. mellonella* larvae model, a mortality rate of 70% was observed upon infection by *K. pneumoniae* ST258 and ST23. This number was reduced to 20% upon treatment with bacteriophages at a multiplicity of infection of 10. This work increases the number of characterized bacteriophages infecting *K. pneumoniae* and provides information regarding genome sequence and efficacy during preclinical phage therapy against two prominent sublineages of this bacterial species.

*Klebsiella pneumoniae*, a member of the Enterobacteriaceae family, causes a variety of human and animal infections including pneumonia, infections of the urinary tract, bacteremia, and liver abscess. *K. pneumoniae* infections are becoming increasingly difficult, and sometimes impossible [1], to treat due to the continuous emergence of multidrug-resistant strains [2,3,4]. Cells of *K. pneumoniae* are characteristically surrounded by a thick capsule of variable chemical composition, which translates into a large number of classically defined capsular serotypes [5] and an even larger number of in silico-defined *wzi*, *wzc,* or KL-types [6,7]. These three molecular classifications denote the diversity of the capsular polysaccharide synthesis gene cluster and serve as proxies of capsular antigen variation. *K. pneumoniae* isolates can be roughly classified into two pathotypes: opportunistic *K. pneumoniae*, which are often multidrug-resistant (mdrKp), and hypervirulent *K. pneumoniae* (hvKp) [8,9], which are able to infect healthy individuals and cause invasive infections including pyogenic liver abscess. The majority of clinical mdrKp and hvKp isolates are part of a small number of genetic lineages (also called clonal groups). Prominent lineages include mdrKpST258, which is frequently associated with specific carbapenemases (i.e., those of the KPC family) and resistant to multiple other antimicrobials, and the ST23 lineage, which is the most frequent cause of liver abscess [1] and can also acquire clinically significant antibiotic resistance genes [10]. Recently, there has been has a sharp increase in the clinical significance of mdrKp and hvKp infections [4,9,11].

New therapeutic strategies are critically needed against *K. pneumoniae* infections. Phage therapy is increasingly recognized as an attractive approach [12]. Previous work has shown that bacteriophages (phages) against *Klebsiella* can be readily isolated from diverse sources and are a promising tool against *K. pneumoniae* infections in *Galleria mellonella* models [13,14].

The aim of this study was to contribute to developments of the phage therapy approach against *K. pneumoniae* and, more specifically, against its two prominent lineages ST23 and ST258. Specifically, our objectives were (i) to isolate and characterize phages against bacteria in these lineages and to sequence the genome of these phages; (ii) to implement an infection model of *G. mellonella* larvae with *K. pneumoniae* strains of interest; and (iii) to test phages against *K. pneumoniae* in this model.

Two clinical *K. pneumoniae* strains were selected for phage isolation [6,15,16]. The first was the 2198 (SB4551) strain, a *K. pneumoniae* carbapenemase-producing isolate from an outbreak in Ireland [15]. This strain, characterized by *wzc*-921 and *wzi*-29 alleles, belongs to ST258 clade 1 [17,18] or ST258a [19] associated with the production of a newly described capsular polysaccharide [20]. It carries *bla*_KPC-2_ and *bla*_TEM-1_ genes, as well as a chromosomal *bla*_SHV-11_ gene; aminoglycoside resistance genes *aac6-Ib* and *aadA2*; mutations in the QRDR region of quinolone targets (ParC-80I, GyrA-83I); genes conferring resistance to phenicols, sulfonamide, tetracycline, and trimethoprim (*catA1, sulI, tetB, dfrA12*); and has no virulence genes. The second strain was SA12 (SB4385), an ST23, K1 capsular-type isolate from a human liver abscess infection in France [9]. It carried virulence genes for yersiniabactin (*ybt 1*; *ICEKp10*), colibactin (*clb 2*), aerobactin (*iuc 1*), salmochelin (*iro 1*), and the regulator of mucoid phenotype genes *rmpA* and *rmpA2*; it has no resistance genes except for the chromosomal gene *bla*_SHV-11_. Phages vB_KpnP_KL106-ULIP47 and vB_KpnP_KL106-ULIP54 were isolated against 2198 and phage vB_KpnP_K1-ULIP33 was isolated against SA12; all three from wastewater collected in France (Clichy, Saint-Denis, and Rueil-Malmaison, respectively) in 2015 using standard procedures [21]. Briefly, the wastewater samples were centrifuged at 4000 rpm for 10 min to remove large particles, then filtered and sterilized (0.45 µm). A first enrichment step was performed at 37 °C for 24 h with gentle agitation (50 rpm). When a clarification of the medium was observed, it was then centrifuged at 5000 *g* for 10 min and 20 µL of supernatant was spread on the surface of LB agar and then covered by a liquid culture of the target bacteria. After incubation for 18 h at 37 °C, individual plaques were selected and purified three times following the same procedure. These three phages produced large, clear plaques surrounded by a halo zone (Figure S1) reflecting the potential presence of an exopolysaccharide depolymerase [22]. The pH, temperature, storage stability, and the lysis kinetic curves were assessed (Figures S2–S5). The host range of the isolated phages was determined using a set of 23 *Klebsiella* spp. strains representative of diverse species and capsular serotypes (Table S1). Based on standard spot assays [23], the three phages showed specificity for the capsular type of their original bacterial host. vB_KpnP_K1-ULIP33 showed clear lysis specifically against the K1 strains, whereas vB_KpnP_KL106-ULIP47 and vB_KpnP_KL106-ULIP54 were specific for the “undefined” capsular type of their parental strain (KL106, *wzi* 29) (Table S1). This capsular specificity probably reflects the need for phages to first adsorb to and depolymerize the thick capsule. The depolymerases allowing the disruption of the polysaccharide capsule are generally K-type specific in *Klebsiella* [24,25,26].

To analyze the genome of these phages, polyethylene glycol (PEG) precipitation was performed, followed by CsCl density gradient (layers of 1.33, 1.45, 1.50, and 1.70 g/cm^3^) ultracentrifugation (28,000 *g*; 3 h; 4 °C), dialysis using Slide-A-Lyzer dialysis cassettes G2 (Thermo Fisher Scientific Inc., Merelbeke, Belgium) and, finally, DNA extraction [27,28]. A sequencing library was obtained using the NEBNext Ultra DNA kit (New England Biolabs, Ipswich, MA, USA) and sequenced using an Illumina MiSeq instrument equipped with a nanoFlowcell (Illumina MiSeq Reagent Nano Kit v2, Brussels, Belgium, paired-end 2*250 bp reads). After correction of reads (Trimmomatic v0.38) [29], assembly (SPAdes v3.9) [30], and analysis of the genome ends (PhageTerm v1.0.11) [31], the average read coverage depths of the assemblies were 550×, 423×, and 815× for phages vB_KpnP_K1-ULIP33, vB_KpnP_KL106-ULIP47, and vB_KpnP_KL106-ULIP54, respectively. Annotation was performed with the RAST server using the virus domain option [32] followed by manual curation. All genomic data related to this project, including raw Illumina read and GenBank annotation, are available via the NCBI BioProject PRJNA488998. GenBank accession numbers are MK380014 (vB_KpnP_K1-ULIP33), MK380015 (vB_KpnP_KL106-ULIP47), and MK380016 (vB_KpnP_KL106-ULIP54). All three phages carry a linear dsDNA genome with predicted direct repeats, totaling 44,122 bp (vB_KpnP_K1-ULIP33), 41,397 bp (vB_KpnP_KL106-ULIP47), and 41,109 bp (vB_KpnP_KL106-ULIP54). Phage vB_KpnP_K1-ULIP33 has direct repeats of length 163 nt, whereas phages vB_KpnP_KL106-ULIP47 and vB_KpnP_KL106-ULIP54 have direct repeats of 180 nt. Comparative genomics of vB_KpnP_K1-ULIP33 with Enterobacteria phage Sp6, and of vB_KpnP_KL106-ULIP47 and vB_KpnP_KL106-ULIP54 with *Klebsiella* phage KP32, illustrate their genetic relatedness to reference phages [33,34] and the conserved genome organization of the *Autographivirinae* subfamily (Figure 1a–c). Distinguishing features of this phage subfamily include a unidirectional and progressive transcriptional scheme, regulated by the presence of a single subunit RNAP driving the middle/late expression. Analysis of the tailspike proteins with HMMER and HHPRED suggested the presence of tailspike-associated depolymerases present in phages vB_KpnP_KL106-ULIP47 (locus D3A56_0040) and vB_KpnP_KL106-ULIP54 (locus D3A57_0040), consistent with the presence of expanding halos in the plaques [35]. These proteins typically show a conserved (T7-related, gp17) N-terminal connector (aa1–154 pfam03906) and diverse C-terminal domains, associated with predicted pectate lyase domains. Pectate lyase domains were previously shown to have depolymerase activity against *Acinetobacter baumannii* polysaccharide capsules and against extracted exopolysaccharides [36,37]. These domains are likely associated with the capsular specificity of these phages [38,39]. Although vB_KpnP_K1-ULIP33 also induced a halo zone around the clear region of plaque lysis, suggestive of a putative depolymerase activity, no depolymerase domain was predicted. However, a tailspike protein (locus D3A55_0041) was found to have a conserved N-terminal phage_T7 connector domain (aa3–171 pfam03906). No gene related to phage lysogeny was predicted, suggesting that these phages are strictly lytic, which is an important prerequisite for phage therapy [40]. The location of the lysis cassette genes in vB_KpnP_KL106-ULIP47 and vB_KpnP_KL106-ULIP54 suggests a typical T7-related genome organization in which the endolysin is located among the middle genes, presumably having a secondary function as a regulator of the phage-encoded RNAP.

To assess the potential in vivo efficacy of phages against *K. pneumoniae* in a preclinical setting with an emphasis on the prevention of infection, a *G. mellonella* larvae model was used. This model allows testing phages within a more complex system than Petri dishes and has interesting features, including similarities between the systemic cellular and humoral immune responses of these larvae and the inflammatory responses of the mammalian innate immune system [41]. Previous reports have found this model to be useful for studies of the virulence of *K. pneumoniae* and for therapeutic approaches [14,42,43,44]. We first determined that the optimal inoculum concentration for *K. pneumoniae* infection was 10^4^ CFU/10 µL, as this dose induced a mortality rate of 70–90% in 4 days, both for strain 2198 and for strain SA12. We confirmed (data not shown) that the mortality of larvae infected with *K. pneumoniae* was dose-dependent [43]. We next assessed phage efficacy against *K. pneumoniae* infection in two independent experimental setups.

In the first experiment, we assessed the efficacy of phage vB_KpnP_K1-ULIP33 against infection by strain SA12. A total of 150 larvae were divided into five groups of 10 larvae with technical triplicates (Table S2a). In a second experiment, we analyzed the individual or combined effect of phages vB_KpnP_KL106-ULIP47 and vB_KpnP_KL106-ULIP54 on strain 2198. Here, a total of 330 larvae were divided into five groups and 11 subgroups of 10 larvae in technical triplicates (Table S2b). In both experiments, phages were administered either 1 h prior to bacterial infection (group A) or 1 h post-bacterial inoculation (group B). The timing of phage inoculation was selected in order to allow the spread of bacteria within the larvae but without allowing enough time for the infection to develop. Groups C, D, and E corresponded to assays of phage toxicity, infectivity control, and injection safety, respectively. Phages were inoculated with a multiplicity of infection (MOI) close to 10 on the left last proleg and the bacterial inoculation was performed on the right last proleg. The concentrations of the inoculated *K. pneumoniae* SA12 and 2198 were verified and were, respectively, 2 × 10^4^ CFU/10 µL and 7 × 10^3^ CFU/10 µL. The titers of the phage inoculums were also verified after inoculation and were 2 × 10^5^ PFU/10 µL for vB_KpnP_K1-ULIP33, 2 × 10^5^ PFU/10 µL for vB_KpnP_KL106-ULIP47, and 7 × 10^4^ PFU/10 µl for vB_KpnP_KL106-ULIP54. Data from each independent experiment were pooled and the protection of the *G. mellonella* larvae by the phages was assessed with the log-rank test (*p*-values < 0.005 were considered as statistically significant). The Kaplan–Meier analyses were performed with the LIFETEST procedure of SAS version 9.4 for Windows and graphs were designed with SAS^®^ ODS Graphics Editor.

Considering the different technical replicates, the first experiment, which tested the in vivo efficacy of vB_KpnP_K1-ULIP33 against SA12, showed that only 0–30% of the larvae survived in the infected groups at 4 days post-inoculation (DPI), whereas the survival rates of prophylactic and treatment groups ranged from 90% to 100%. In the second experiment, which tested the in vivo efficacy of vB_KpnP_KL106-ULIP47 and vB_KpnP_KL106-ULIP54 against strain 2198, 0–10% of the larvae survived in the infected groups at 4 DPI, whereas the survival rates of prophylactic and treatment groups ranged from 80% to 100%. In both experiments, groups of larvae inoculated with phage (but not bacteria) showed comparable survival rates as the PBS control groups, ranging from between 70% and 100% (Figure S1a,b). The survival curves are presented in Figure 2; data from the triplicate experiments were pooled. Protection of the *G. mellonella* larvae by the phages was found to be statistically significant (*p*-values < 0.0001 for each experiment). No significant difference was observed between the cocktail and the monophage groups. Note that despite their different stabilities (Figures S2–S5), these phages have high genetic relatedness and similar host ranges, and may therefore not be the best candidates for a phage cocktail.

These data show that the three studied phages could efficiently prevent a *K. pneumoniae* infection induced by their host strains. Both phage only and PBS control groups showed similar survival rates, demonstrating the safety of the phages in this model. A recent report indicated protection against *K. pneumoniae* ST258 infection in *G. mellonella* with another phage [14]. The present study confirms that strains belonging to this ST can be targeted by phages and reports, for the first time, on phage efficacy against ST23 *K. pneumoniae* in *G. mellonella*. The very low MOI used in this study allowed for assessment of the efficacy of the phages while avoiding the phenomenon of “lysis from without”. Overall, this study confirms that the *G. mellonella* is a flexible and rapid tool to assess phage efficacy. Indeed, it accommodates many human pathogenic strains in contrast to rodent models and it allows a quick (less than 48 h in this study) evaluation of the killing activity of phages in vivo. However, the relevance of the *G. mellonella* model to predict the phage efficacy in higher animals including humans, and in particular, with higher MOIs and timings of phage administration, remains to be determined [44].

## Figures and Tables

**Figure 1:**
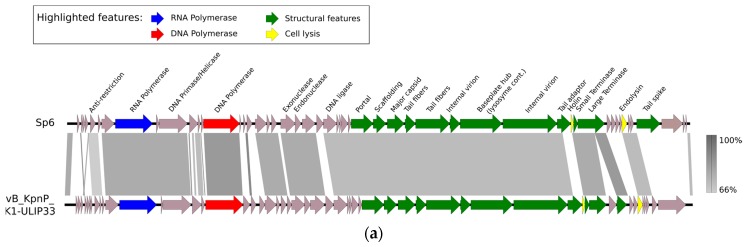

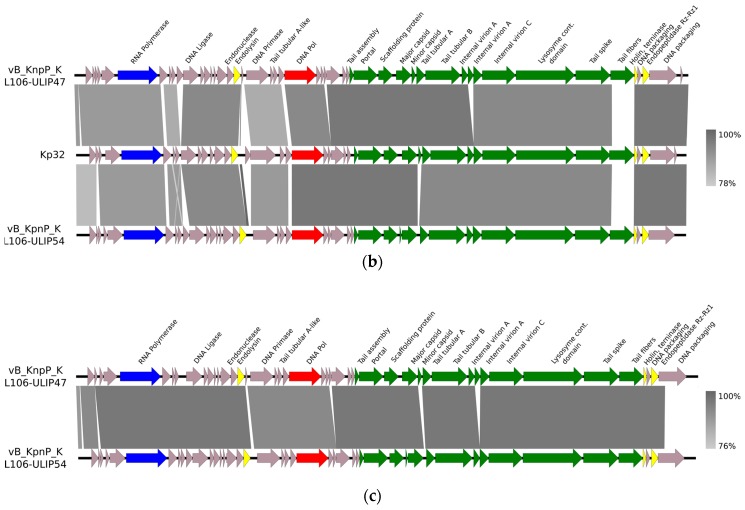
Comparative genomics (nucleic acid sequence) of (**a**) vB_KpnP_K1-ULIP33 with Enterobacteria phage Sp6 (Genus Sp6virus, AY288927), (**b**) vB_KpnP_KL106-ULIP47, vB_KpnP_KL106-ULIP54 with *Klebsiella* phage *Klebsiella pneumoniae* 32 (Genus *K. pneumoniae* 32virus, MH172262); and (**c**) vB_KpnP_KL106-ULIP47 with vB_KpnP_KL106-ULIP54.

**Figure 2:**
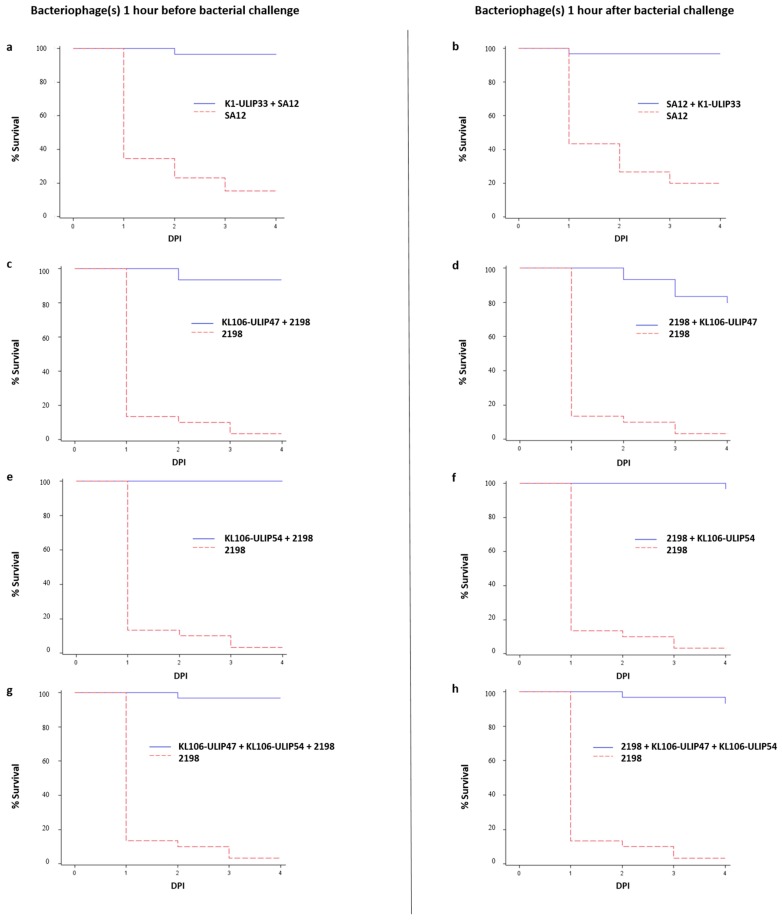
Kaplan–Meier survival curves of the *Galleria mellonella* larvae inoculated with *K. pneumoniae* SA12 (ST23) (**a**,**b**) and *K. pneumoniae* 2198 (ST258) (**c**–**h**) with, respectively, phage vB_KpnP_K1-ULIP33 (K1-ULIP33), and phages vB_KpnP_KL106-ULIP47 (KL106-ULIP47) and vB_KpnP_KL106-ULIP54 (KL106-ULIP54), one hour before or one hour after bacterial inoculation.

## Supplementary Materials

The following are available online at https://www.mdpi.com/1999-4915/11/5/411/s1, Figure S1: Picture of halo zones of Phages (a) vB_KpnP_K1-ULIP33, (b) vB_KpnP_KL106-ULIP47 and (c) vB_KpnP_KL106-ULIP54. Figure S2: The temperature stability of phages vB_KpnP_K1-ULIP33 (A), vB_KpnP_KL106-ULIP47 (B), and vB_KpnP_KL106-ULIP54 (C). Figure S3: The pH stability of phages vB_KpnP_K1-ULIP33 (A), vB_KpnP_KL106-ULIP47 (B), and vB_KpnP_KL106-ULIP54 (C). Figure S4: The storage stability of phages vB_KpnP_K1-ULIP33 (A), vB_KpnP_KL106-ULIP47 (B), and vB_KpnP_KL106-ULIP54 (C) at 4 °C. Figure S5: Lysis kinetic curves of vB_KpnP_K1-ULIP33 lysis on the SB4385 strain (A), vB_KpnP_KL106-ULIP47 (B), and vB_KpnP_KL106-ULIP54 (C) on the SB4551 strain. Table S1: Bacterial strains characteristics and phages spot assays results. Table S2: Experimental designs of the main *Galleria mellonella* experiments with (a) *K. pneumoniae* SA12 (ST23) and phage vB_KpnP_K1-ULIP33 and (b) *K. pneumoniae* 2198 (ST258), phage vB_KpnP_KL106-ULIP47, and vB_KpnP_KL106-ULIP54. Each group contains 10 larvae and each experiment condition was reproduced in technical triplicates.
