# Supplementary material for: New Bacteriophages against Emerging Lineages ST23 and ST258 of Klebsiella pneumoniae and Efficacy Assessment in Galleria mellonella Larvae
Source: Viruses. 2019 May 3;11(5):411. doi: 10.3390/v11050411 (PMC6563190; doi:10.3390/v11050411)
Supplement: Supplementary file 1 [file viruses-11-00411-s001.pdf]

SUPPLEMENTARY MATERIALS

(a)

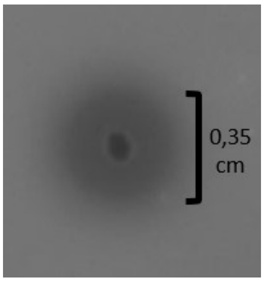

(b)

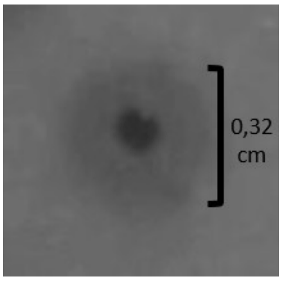

(c)

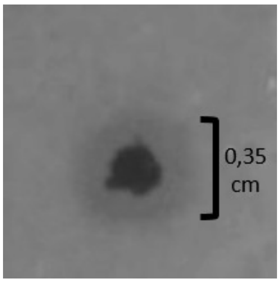

Figure S1. Picture of halo zones of phages (a) vB_KpnP_K1-ULIP33, (b) vB_KpnP_KL106-ULIP47 and (c) vB_KpnP_KL106-ULIP54.

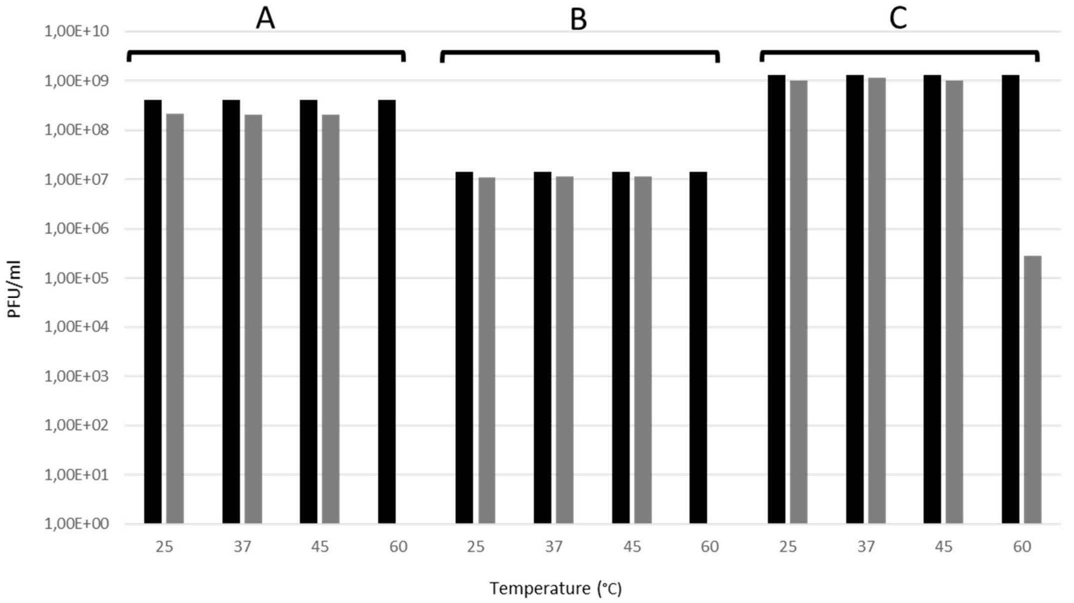

Figure S2. The temperature stability of phages vB_KpnP_K1-ULIP33 (A), vB_KpnP_KL106-ULIP47 (B) and vB_KpnP_KL106-ULIP54 (C). Each grey bar represents the mean of three independent experiments (biological triplicates) (Black bar = initial titer; Grey bar = titer after 1 hour of heat treatment).

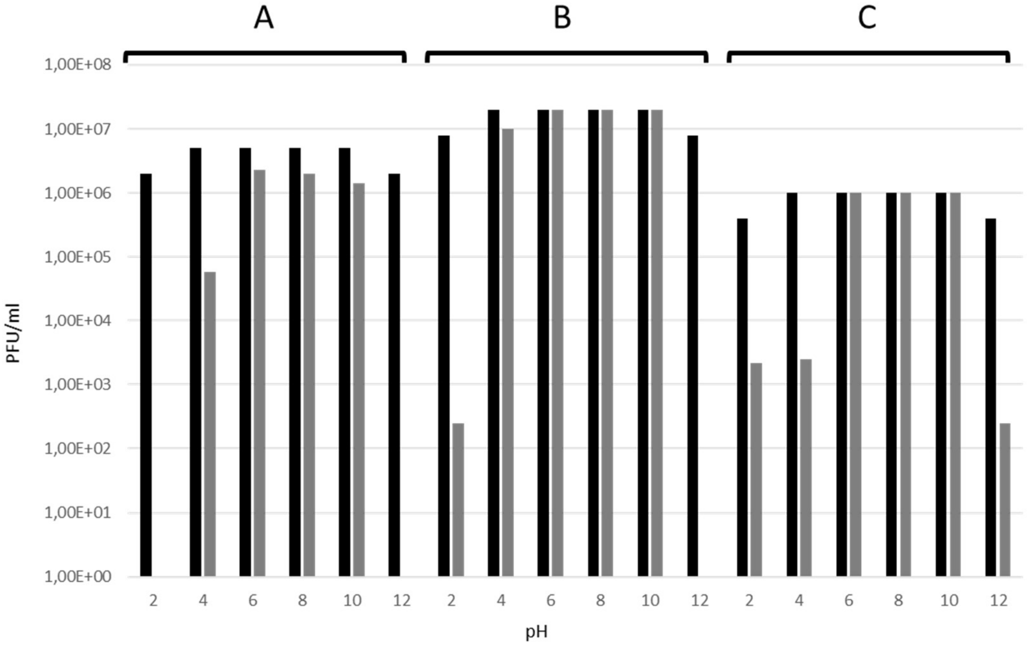

Figure S3. The pH stability of phages vB_KpnP_K1-ULIP33 (A), vB_KpnP_KL106-ULIP47 (B) and vB_KpnP_KL106-ULIP54 (C). Each grey bar represents the mean of three independent experiments (biological triplicates) (Black bar = initial titer; Grey bar = titer after 1 hour of pH treatment at 37°C).

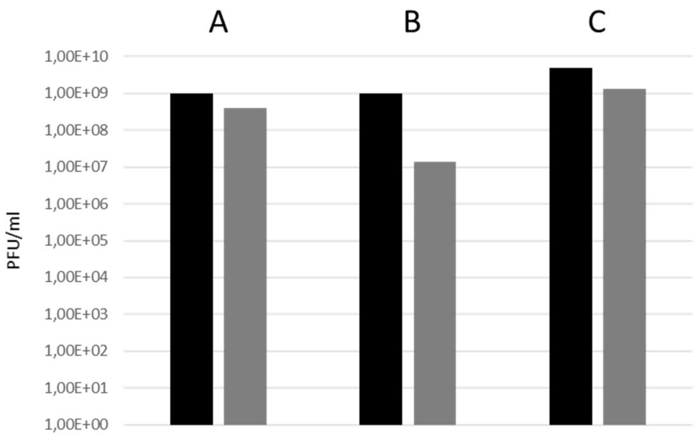

Figure S4. The storage stability of phages vB_KpnP_K1-ULIP33 (A), vB_KpnP_KL106-ULIP47 (B) and vB_KpnP_KL106-ULIP54 (C) at 4°C. Each grey bar represents the mean of three experiments (technical triplicates) (Black bar = initial titer; Grey bar = titer after 2 years at 4°C).

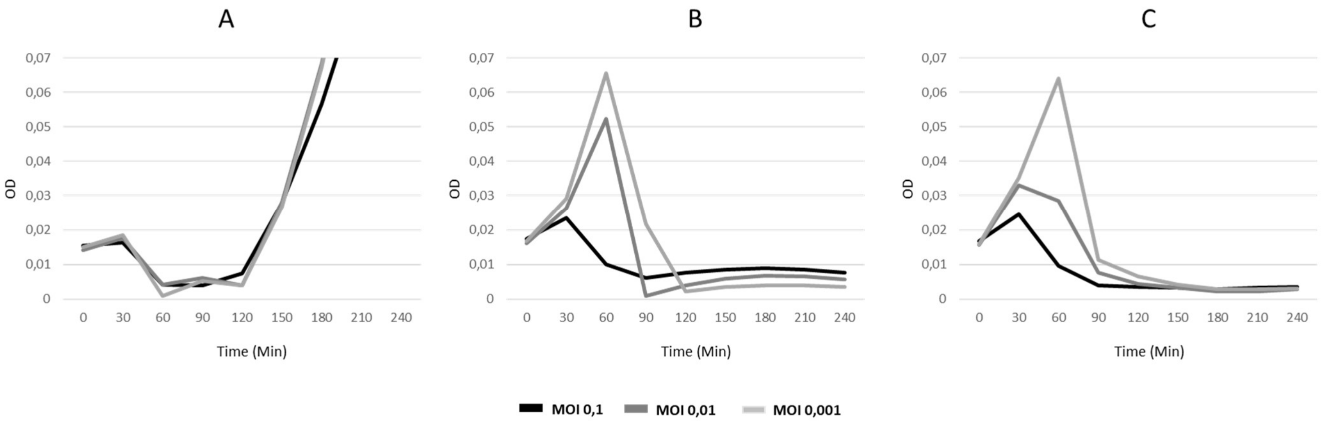

Figure S5. Lysis kinetic curves of vB_KpnP_K1-ULIP33 lysis on the SB4385 strain (A), Kunk-ULIP47 (B) and Kunk-ULIP54 (C) on the SB4551 strain. Each bar represents the mean of three independent experiments (biological triplicates).

Table S1. Bacterial strains characteristics and bacteriophages spot assays results.

ND: not determined; /: no lysis; OL: opaque lysis; OL: clear lysis; PS: Propagating strain.

Strain characteristics								Spot tests		
**ST**	**Capsular type**	**KL type**	**Strain**	**Species**	**Source**	***wzc* type**	***wzi* type**	**vB_KpnP_KL106-ULIP47**	**vB_KpnP_KL106-ULIP54**	**vB_KpnP_K1-ULIP33**
**23**	K1	1	SA12	*K. pneumoniae*	Virulent, community	905	1	/	/	CL (PS)
**23**	K1	1	NTUH-K2044	*K. pneumoniae*	Virulent, community	1	1	/	/	/
**258**	ND	106	2198	*K. pneumoniae*	Clinical, MDR	921	29	CL (PS)	CL (PS)	OL
**14**	K2	2	cur15505	*K. pneumoniae*	Clinical, MDR	2	2	/	/	/
**15**	K24	24	04A025	*K. pneumoniae*	Clinical, MDR	25	24	/	/	/
**17**	K2	2	SB4-2	*K. pneumoniae*	Carrier status	2	186	/	/	/
**37**	K8	8	SB1139	*K. pneumoniae*	Carrier status	8	8	/	/	OL
**38**	K52	52	MGH 78578	*K. pneumoniae*	Clinical, MDR	51	50	/	/	/
**45**	K24	24	SB1170	*K. pneumoniae*	Carrier status	ND	101	/	/	/
**55**	ND	124	SB617	*K. pneumoniae*	Environment	938-like	447	/	/	/
**62**	ND	124	SB615	*K. pneumoniae*	Environment	ND	447	/	/	/
**65**	K2	2	SB3332	*K. pneumoniae*	Virulent, community	2	157	/	/	OL
**66**	K2	2	CIP 52.145	*K. pneumoniae*	Virulent, community	2	4	/	/	OL

**67**	K3	3	SB3432	*K. pneumoniae*	Clinical, rhinoscleroma	ND	132	/	/	OL
**86**	K2	2	SA1	*K. pneumoniae*	Virulent, community	2	2	/	/	OL
**90**	4	107	SB3464	*K. pneumoniae*	Clinical, laryngeal scleroma	71	127	/	/	OL
**133**	ND	116	SB612-2	*K. pneumoniae*	Environment	ND	180	/	/	/
**375**	K2	2	SB4536	*K. pneumoniae*	Virulent, community	2	72	/	/	OL
**380**	K2	2	SB4496	*K. pneumoniae*	Virulent, community	2	2	/	/	/
**1215**	K53	53	07A044	*K quasipneumoniae* subsp. *similipneumoniae*	Clinical, MDR	52	164	/	/	OL
**1528**	K35	35	SB11	*K. quasipneumoniae* subsp. *quasipneumoniae*	Clinical, MDR	ND	15	/	/	/
**2273**	K31	31	01A065	*K. variicola*	Clinical, MDR	934	32	/	/	/
**2668**	ND	111	SB611	*K. pneumoniae*	Environment	940	563	/	/	OL

Table S2. Experimental designs of the main Galleria mellonella experiments with (a) *K. pneumoniae* SA12 (ST23) and bacteriophage vB_KpnP_K1-ULIP33 and (b) *K. pneumoniae* 2198 (ST258), bacteriophage vB_KpnP_KL106-ULIP47 and vB_KpnP_KL106-ULIP54. Each group contains 10 larvae and each experiment condition was reproduced in technical triplicates.

a)

Injection time (H)	*Group*				
	A	B	C	D	E
-1	K1-ULIP33	/	/	/	/
0	SA12		K1-ULIP33	SA12	PBS
+1	/	K1-ULIP33	/	/	/

b)

Injection time (H)	***Group***										
	A1	A2	A3	B1	B2	B3	C1	C2	C3	D	E
-1	KL106-ULIP47	KL106-ULIP54	KL106-ULIP47 KL106-ULIP54	/	/	/	/	/	/	/	/
0	2198						KL106-ULIP47	KL106-ULIP54	KL106-ULIP47 KL106-ULIP54	2198	PBS

+1	/	/	/	KL106-ULIP47	KL106-ULIP54	KL106-ULIP47<br>KL106-ULIP54	/	/	/	/	/
